# Supplementary material for: Rheology of rounded mammalian cells over continuous high-frequencies
Source: Nat Commun. 2021 May 18;12:2922. doi: 10.1038/s41467-021-23158-0 (PMC8131594; doi:10.1038/s41467-021-23158-0)
Supplement: Supplementary file 1 — Supplementary Information [file 41467_2021_23158_MOESM1_ESM.pdf]

SUPPLEMENTARY INFORMATION

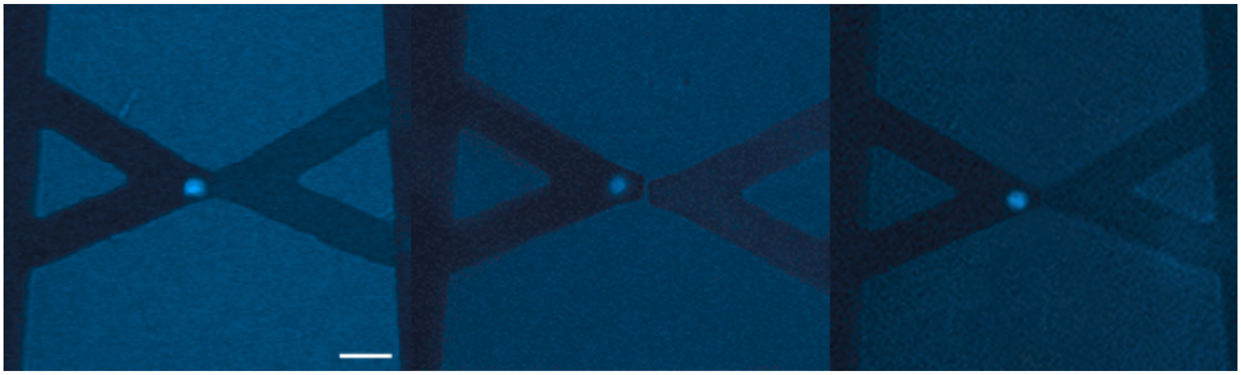

Supplementary Figure 1 | Rheological measurements do not compromise cell viability. After rheological characterization, the HeLa cell was retracted from the slave cantilever. Following a waiting time of three hours the markers NucBlue® live reagent (Hoechst 33342) and NucGreen® dead reagent from the ReadyProbes™ Cell Viability Imaging Kit (Thermofischer) were added and the cell was imaged by fluorescence microscopy. The presence of the fluorescent signal of NucBlue® and the absence of NucGreen® indicate a viable cell. The experiment was repeated three independent times. Scale bar, 20 µm.

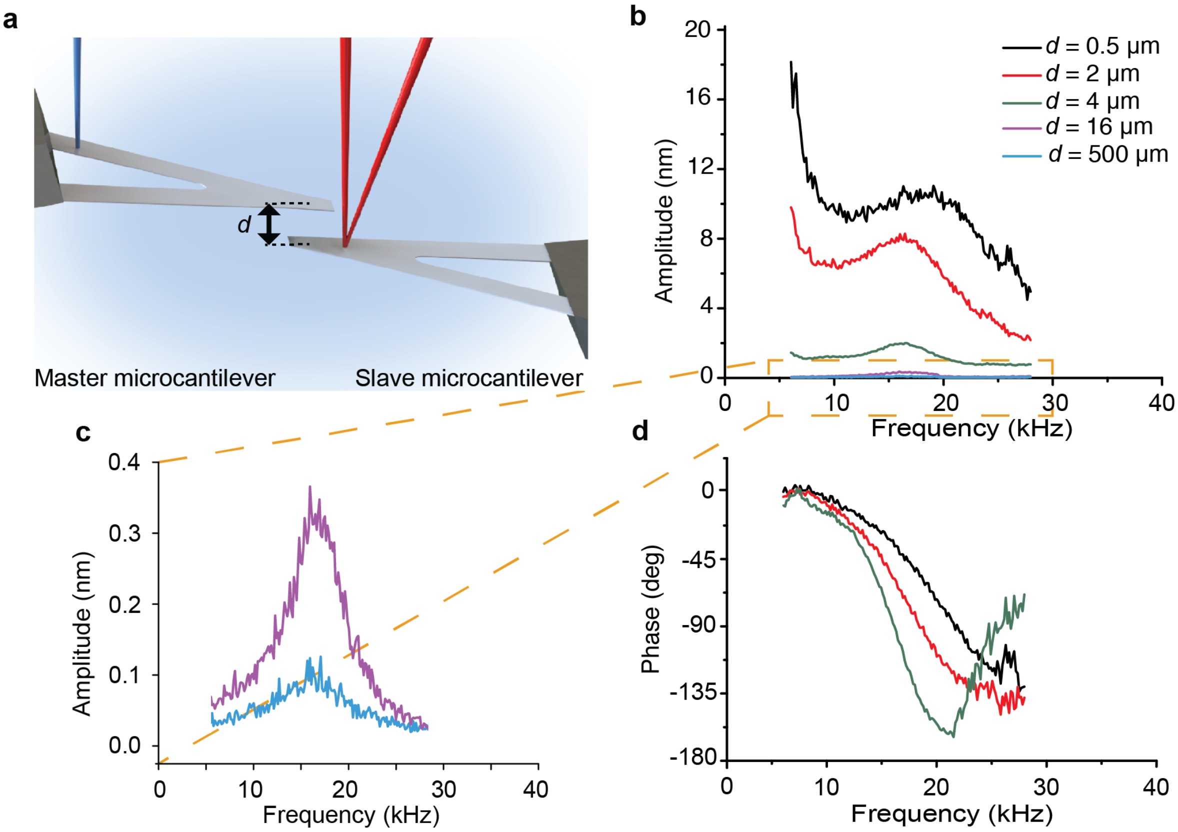

Supplementary Figure 2 | The distance-dependent water-mediated coupling of two microcantilevers is negligible for cantilever separations similar to the cell size. a, Experimental setup used to measure the mechanical coupling between the parallel (sandwiching) master and slave microcantilevers (**Fig. 1**) in the absence of a cell. Both microcantilevers were kept separated by a variable distance d. The master microcantilever oscillated with an amplitude of 13 nm at its resonance (≈ 18 kHz), which we also applied to characterize cell mechanical properties (**Methods**). **b,** Amplitude spectrum of the slave microcantilever when actuating the master cantilever at separations of d = 0.5, 2, 4, and 16 µm as well as $d = 500\ \mu\text{m}$, the latter distance was used to measure the thermal noise of the slave cantilever. For small separations of ≤ 2 µm the slave cantilever amplitude is high, however, it drops by more than a decade (to ≈ 4% of the amplitude of the master cantilever) for the separation of 16 µm, which corresponds to the average diameter of a rounded HeLa cell. **c,** Blow-up of the slave cantilever amplitude spectrum for $d = 16\ \mu\text{m}$ and $d = 500\ \mu\text{m}$. The oscillation amplitude of the slave microcantilever triples (violet) compared to the thermal noise (light blue), which indicates an energy transfer of the master cantilever to the slave cantilever *via* the water. However, the amplitude of the slave microcantilever in the absence of a cell (violet) is more than a decade smaller than the amplitudes in the presence of a living cell sandwiched between both microcantilevers (**Fig. 1**). Together with **Fig. 1**, the experiment shows that the rounded HeLa cell sandwiched between both microcantilevers dominates the mechanical transfer from the master microcantilever to the slave microcantilever. **d,** Phase spectra of the slave microcantilever being actuated by a master microcantilever at d = 0.5 µm (black), 2 µm (red), and 4 µm (green). With increasing distance, the water-mediated coupling between both cantilevers gets smaller, which results in a lower amplitude (**a**) and in a higher phase delay. As the phase cannot be measured accurately for small amplitudes (see green spectrum above 21 kHz), the measurement bandwidth is reduced compared to measurements sandwiching a cell between both microcantilevers. For the same reason, the phase of the slave cantilever cannot be measured at $d = 16\ \mu\text{m}$. A phase does not exist in the thermal noise case ($d = 500\ \mu\text{m}$).

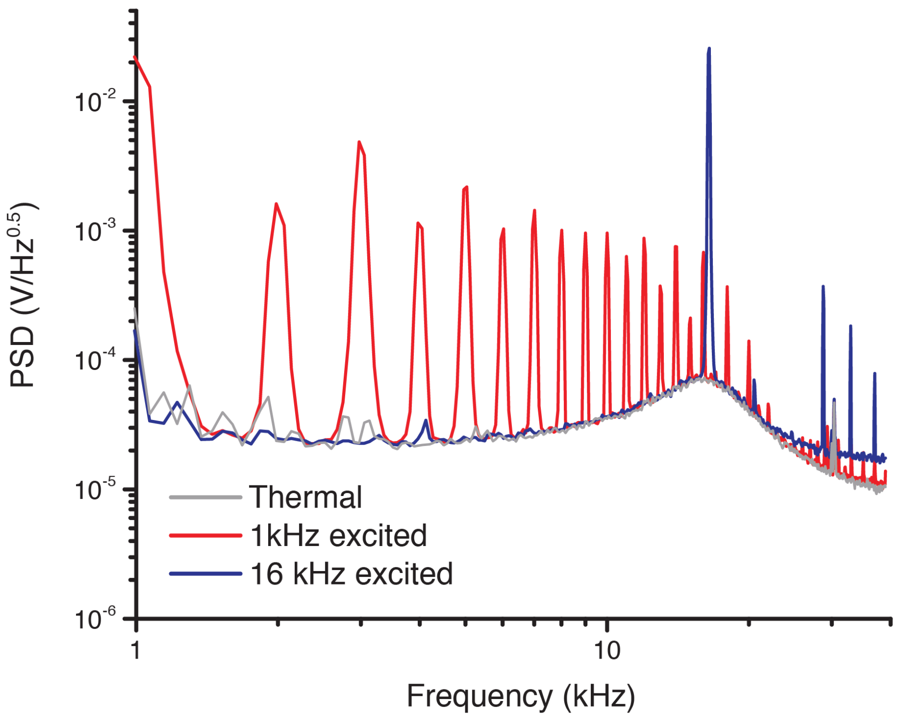

Supplementary Figure 3 | Lock-in amplification allows for low noise detection of the measured signal. The power spectral noise density (PSD) of a single cantilever was recorded with a lock-in amplifier under three actuation conditions: i) thermal noise (no photothermal actuation, black data), ii) photothermal actuation at a frequency of 1 kHz (red data), and iii) photothermal actuation at a frequency of 16 kHz (blue data). The average laser power of 3.5 mW was the same for both frequencies. The PSD at the frequencies of actuation is 200 times higher compared to the thermal PSD, therefore rendering the contribution of the thermal noise negligible for our rheological measurements and allowing for a high data acquisition speed, as averaging multiple measurements is not necessary. The Fourier components at multiples of the excitation frequency of the blue laser, arise due to a slight asymmetry of the sinusoidal movement of the photoactuated cantilever, which is caused by thermal effects.

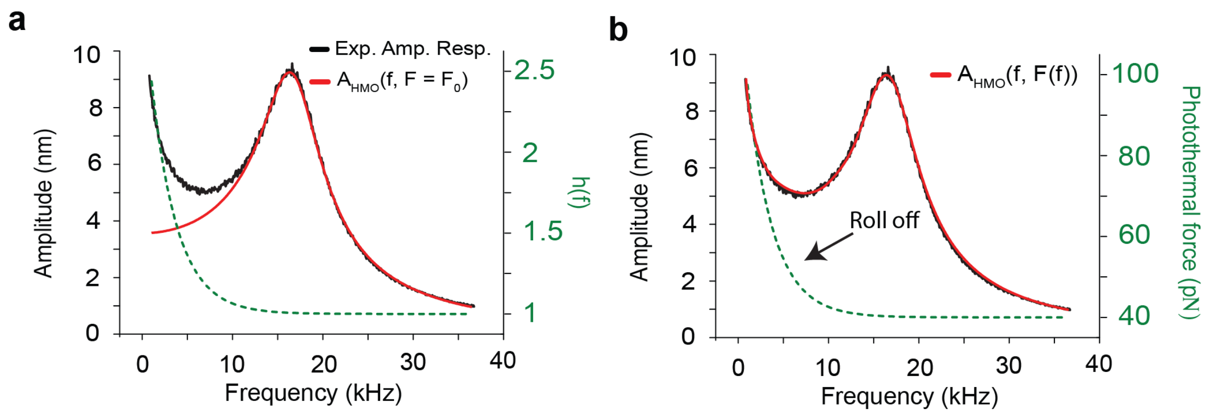

Supplementary Figure 4 | Frequency-dependent photothermal actuation force of the microcantilever. **a,** The experimentally recorded amplitude response of a photothermally actuated microcantilever (black line) is fitted using the amplitude $A_{\mathrm{HMO}}(f, F = F_0) = \frac{1}{4\pi^2}\frac{F_0/m^*}{\sqrt{\left(f_{\mathrm{cant}}^2 - f^2\right)^2 + (f_{\mathrm{cant}}f/Q)^2}}$ of a damped harmonic oscillator (red line) having an effective mass m*, a quality factor Q, and a natural resonance frequency f_{cant}, and being actuated by a constant force F_0. Dividing the experimental amplitude response by A_{HMO}, results in the scaling factor $h(f)$ (green dashed line). **b,** Multiplication of F_0 with the scaling factor function $h(f)$ gives the frequency-dependent actuation force $F(f)$ at which A_{HMO} matches the experimental amplitude response (red line). As predicted by the theory of photothermal actuation[1], $F(f)$ increases at lower frequencies.

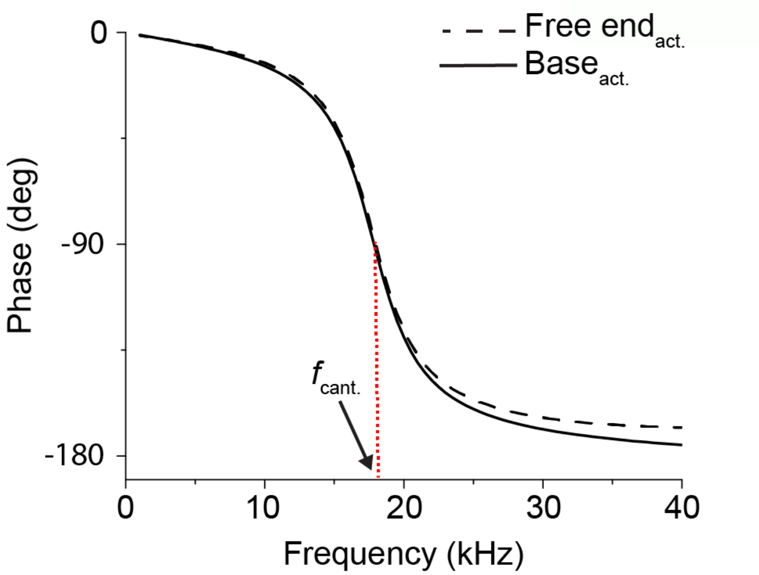

Supplementary Figure 5 | Shifting the position of the photothermally actuating laser on the master microcantilever changes its mechanical response. Shown are finite elements-simulations (**Methods**) of the phase of a microcantilever, which is either photothermally actuated at the base (continuous curve) or the free end (dashed curve). At actuation frequencies below the resonance frequency of the microcantilever $f_{\mathrm{cant}} \approx 18.5$ kHz, the difference in phase is negligible. However, deviations occur above the resonance frequency of the microcantilever. To apply Eq. 1, which is an implicit assumption of the lumped-mass model (**Fig. 2b**), such deviations have to be corrected. Accordingly, to account that the blue photothermally actuating laser and the red readout laser are not at the same positions, the position of the actuating blue laser is mathematically shifted (**Supplementary Fig. 6**).

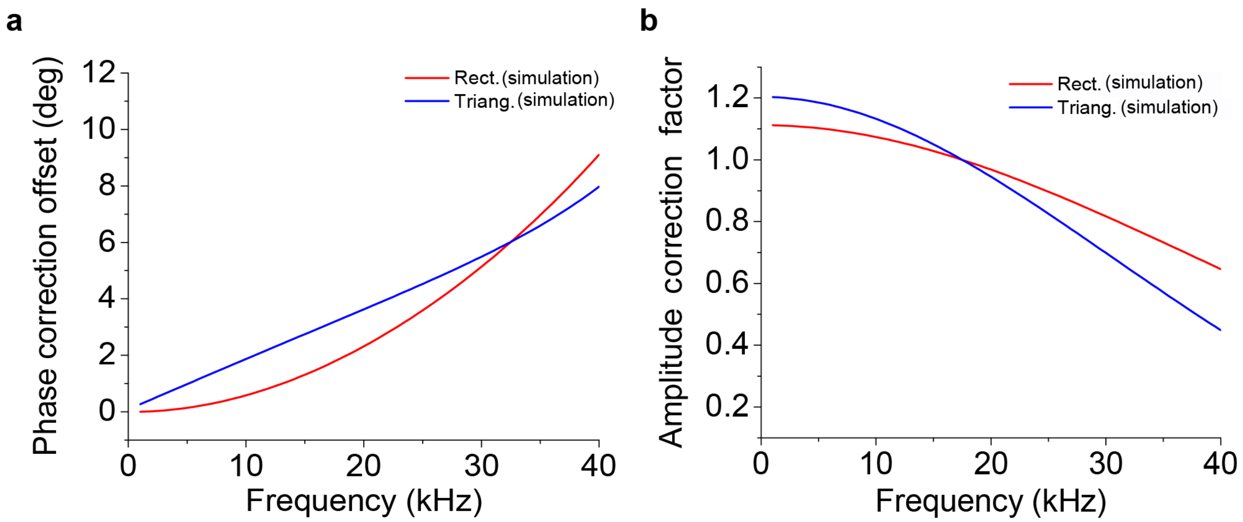

Supplementary Figure 6 | Phase and amplitude correction curves to account for the blue laser position on the photothermally actuated microcantilever. a, Correction curves to transform the phase response of rectangular (red) and triangular (blue) microcantilevers. Addition of the phase correction offset to the experimental phase mathematically shifts the position of the photothermally actuating force, which is exerted by the blue laser (**Methods**). **b,** Correction curves to transform the amplitude response of rectangular and triangular microcantilevers. Eq. 1 does not account for the blue actuation laser and the red readout laser being at different positions on the microcantilevers. Transforming the measured data using the correction curves does this. The correction curves are calculated from the difference (phase) or the ratio (amplitude) of the curves simulated in **Fig. 2c** and **Supplementary Fig. 5**. Applying the corrections to the experimentally measured data, virtually shifts the position of the oscillating force applied by the photothermally actuating blue laser. In the example shown the position of the photothermally actuating laser shifts from the free end ($z = 110$ μm) to the base ($z = 20$ μm). Calculations to mathematically shift other positions of the photothermally actuating laser are applied accordingly (not shown).

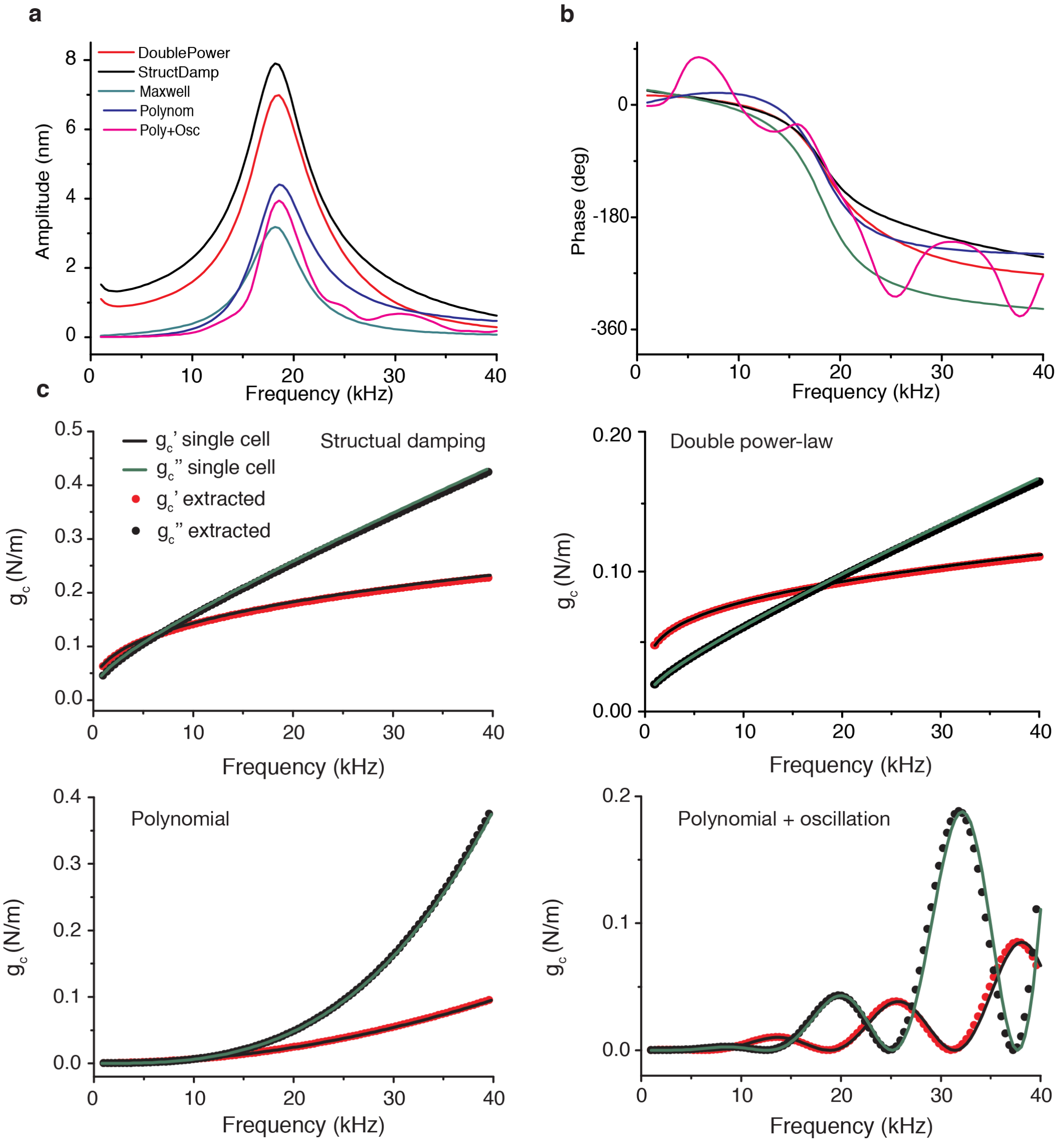

Supplementary Figure 7 | The mechanical de-embedding framework functions independently of the cell mechanical properties. a, **b,** Amplitude and phase response curves of the slave cantilever extracted from finite elements (FEM) simulations in the MS configuration (**Fig. 2**). To generate the data, the same geometries of a rounded cell sandwiched between two triangular cantilevers, and of the cantilever driving force were used, however, the dependencies of the complex modulus E^*_{cort} on the cantilever oscillation frequency f differed as following:

1) Double power-law model: $E^*_{\mathrm{cort}}(f) = A(if/f_0)^{\alpha} + B(if/f_0)^{\beta}$ $A = 2.5\ \mathrm{kPa},\ \alpha = 0.2,\ B = 0.21\ \mathrm{kPa}, \beta = 0.94,\ f_0 = 1\ \mathrm{kHz}$

2) Structural damping model: $E^*_{\mathrm{cort}} = E_{\mathrm{s}}(f/f_0)^{\kappa-1}\big(1 + i\Omega(\kappa)\big)\Gamma(2-\kappa)\cos\pi/2(\kappa-1)\ + if\eta_s$, with $\Omega(\kappa) = \tan(\kappa-1)\,\pi/2, E_{\mathrm{s}} = 2.7\ \mathrm{kPa},\ f_0 = 1\ \mathrm{kHz}, \kappa = 1.35, \eta_s = 360\ \mathrm{Pa \cdot s}$

3) Maxwell model: $E^*_{\mathrm{cort}} = 1/(E_{\mathrm{m}}\ - i/(f\eta_{\mathrm{m}}))$ with $E_{\mathrm{m}} = 10\ \mathrm{kPa}, \eta_{\mathrm{m}} = 0.007s$

4) Polynomial function: $E^*_{\mathrm{cort}} = C(f/f_0)^c + iD(f/f_0)^d$ with $C = \frac{3\mathrm{Pa}}{\mathrm{s}^2}, c = 2, D = 1.5\frac{\mathrm{Pa}}{\mathrm{s}^3}, d = 3$

5) Polynomial + oscillation function: $E^*_{\text{cort}} = C(f/f_0)^c \cos(f/4f_0)^2 + iD(f/f_0)^d \sin(f/4f_0)^2$

The FEM simulation was done using the solid mechanics module of Comsol 5.3a. The geometry of the sandwiched cell was set to a contact radius of 5 µm and a height of 12 µm. **c**, Real g'_c and imaginary g''_c spring constants extracted from FEM simulated cantilever amplitude and phase response curves match the underlying properties of the simulated cell. The extraction of the Maxwell Model is shown in **Fig. 2f.**

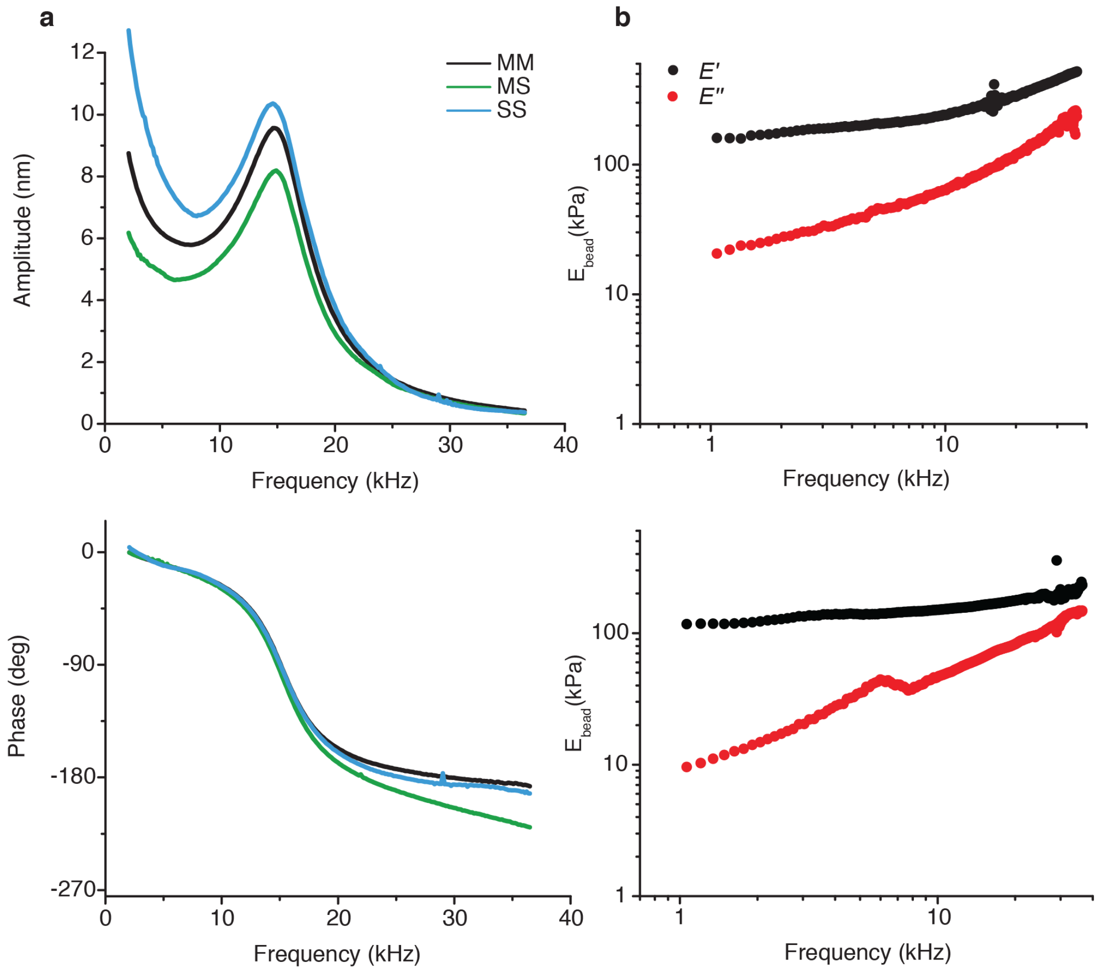

Supplementary Figure 8 | Rheological properties measured of alginate beads agree with literature values. a, Amplitude and phase curves of an alginate bead (2% v/v alginate) sandwiched between master and slave cantilever. The alginate beads had diameters ranging between 20 – 30 µm (n ≥ 20). **b**, Extracted storage E'_{bead} and loss E''_{bead} moduli for two exemplary alginate beads. The characteristic elastic plateau is visible in E'_{bead} (black data points) between 1 kHz and 10 kHz as it has been observed for crosslinked gels[2], to which alginate belongs to in the presence of 10 mM calcium ions ($CaCl_2$) in the buffer solution at 37 °C.

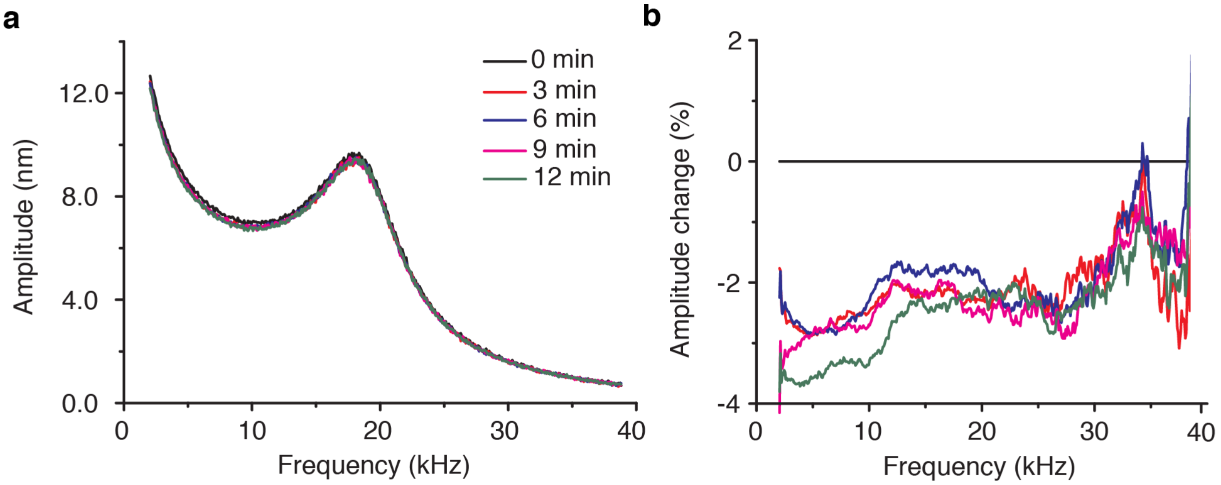

Supplementary Figure 9 | HeLa cells confined between two microcantilevers stabilize within 3 minutes. **a,** Subsequent frequency sweeps recorded of a single rounded HeLa cell compressed by 1 µm using the parallel microcantilever assay (**Fig. 1a-c**). Frequency sweeps were recorded every 3 min. **b,** Amplitude changes among the frequency sweeps. The first sweep recorded at 0 min was set as reference (black). In the second sweep recorded at 3 min, the amplitude reduced by ≈ 2%. The sweeps subsequently recorded at 6, 9 and 12 min showed much smaller amplitude variations compared to the sweep recorded at 3 min (< 1%). Based on this result, we confined single HeLa cells in our parallel microcantilever assay and allowed each cell to relax for 5 min before starting the rheology measurements.

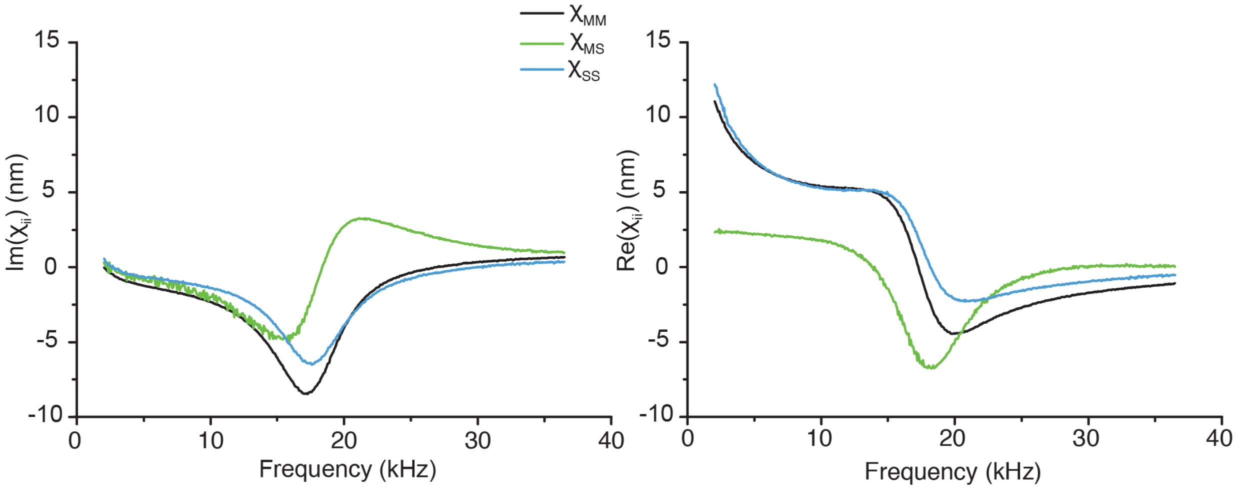

Supplementary Figure 10 | Raw data of the three functions $\chi_{\mathbf{MM}}$, $\chi_{\mathbf{MS}}$, and $\chi_{\mathbf{SS}}$ measured to determine the high-frequency rheology of unperturbed HeLa cells. As the functions are complex-valued, the imaginary (left) and real (right) part are displayed separately. The raw data corresponds to the response curves shown in **Fig. 3a**.

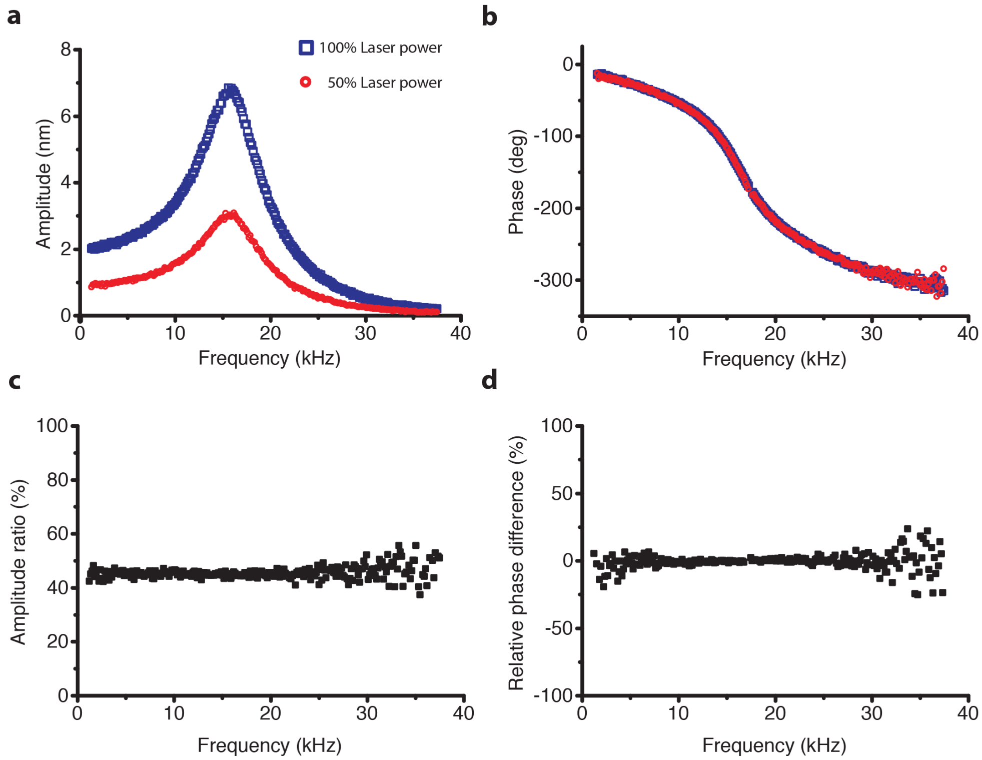

Supplementary Figure 11 | Amplitude and phase responses of a living, unperturbed HeLa cell are linear with respect to the amplitude of the actuating cantilever. a and **b**, Upon reducing the maximum driving amplitude of the actuated master cantilever from 10.6 nm to 4.6 nm (≈ 45% reduction), which results from reducing the power of the actuating laser of 3.5 mW by 50%, the (**a**) amplitude and (**b**) phase response of the slave microcantilever was measured in configuration MS (**Fig. 2a**). The amplitude response of the slave microcantilever shows peak amplitudes of 6.9 nm (3.5 mW laser power) and 3.0 nm (50% of 3.5 mW laser power). **c**, The linear ratio of both amplitude responses measured in (**a**) shows an equal reduction of ≈ 45% for the response amplitude over all frequencies. **d**, Relative phase difference of the slave microcantilever showing a peak amplitude of 4.5 nm (50% laser power) compared to the slave cantilever showing a peak amplitude of 10 nm. The phase change fluctuates around zero over all frequencies. The data scattering at higher frequencies (≈ 30 – 38 kHz) is due to lower amplitudes and, therefore, lower signal-to-noise ratio. Both amplitude ratio and phase change curves show that the mechanical response of the living HeLa cell is linearly proportional to the actuated master cantilever amplitude (**Supplementary Note 1**). It can be reasoned, that the experimentally found linear response of the cell stems from the actuating amplitude of 10 nm, which is a small perturbation compared to the thickness of the cell cortex (≈ 200 nm, **Fig. 4e,f**).

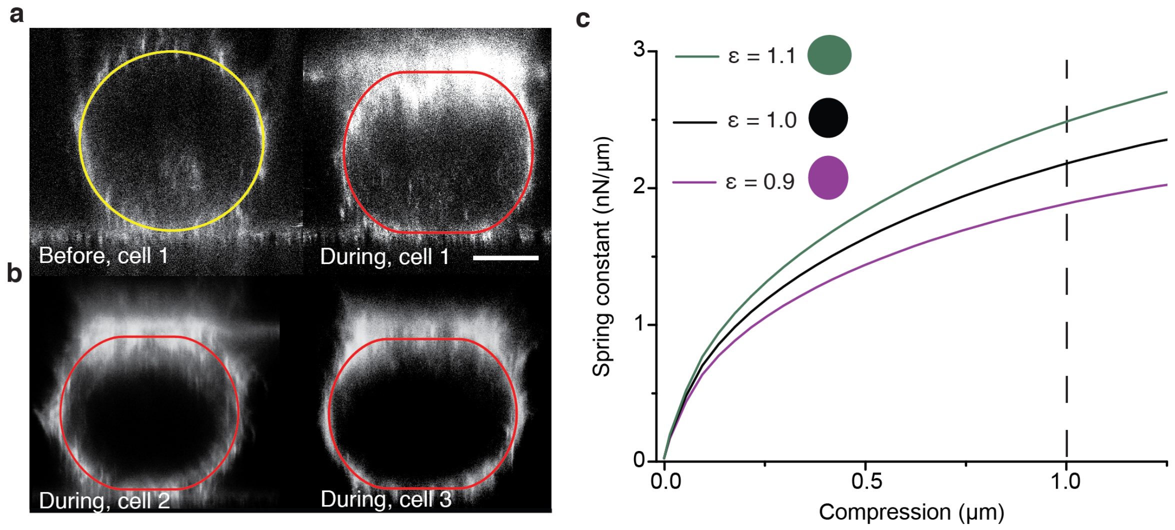

Supplementary Figure 12 | Geometry- of rounded HeLa cells confined between two parallel surfaces agree with the finite elements simulation of the cell shape within the experimental error. a, Sideview of a SiR-actin stained, living unperturbed HeLa cell before (left) and during parallel-plate compression (right). The fluorescence images were recorded using super resolution (stimulated emission depletion, STED) microscopy. The unperturbed cell is outline yellow. The red outline of the compressed cell has been generated from finite elements simulations, which took the experimentally approximated circumference of the unperturbed HeLa cell (yellow) and the amount of compression (≈ 10% of cell height) as input. Due to an optical distortion arising from the gold-coated cantilever reflecting and scattering the lasers used for the STED microscopy, the SiR-actin signal is broadened at the cantilever-cell interface (top). The scale bar is 6 μm. **b**, Two HeLa cells of different size being compressed by our parallel-plate assay. Red lines show outlines from the finite elements simulation for the respective experimental compression of ≈ 1 µm. For parallel-plate compression a wedged microcantilever was pressed onto (top of image) a rounded HeLa cell plated on a Petri dish (bottom of image) under cell culture conditions. **c**, Finite element simulated spring constant of cell geometries with different eccentricities ε. The spring constant depends linearly on ε for a compression ≈ 1 μm, which is used in the experiments. An $\varepsilon = 1.1$ (10% away from an ideal sphere) leads to a spring constant that is ≈ 10% higher than for $\varepsilon = 1.0$ and to an overestimation of the storage and loss moduli by 10%, if the geometry was not correctly accounted (i.e., the cell is assumed spherical). In our experiments, however, only small eccentricities (≈ 1 – 4%) were observed for the cells.

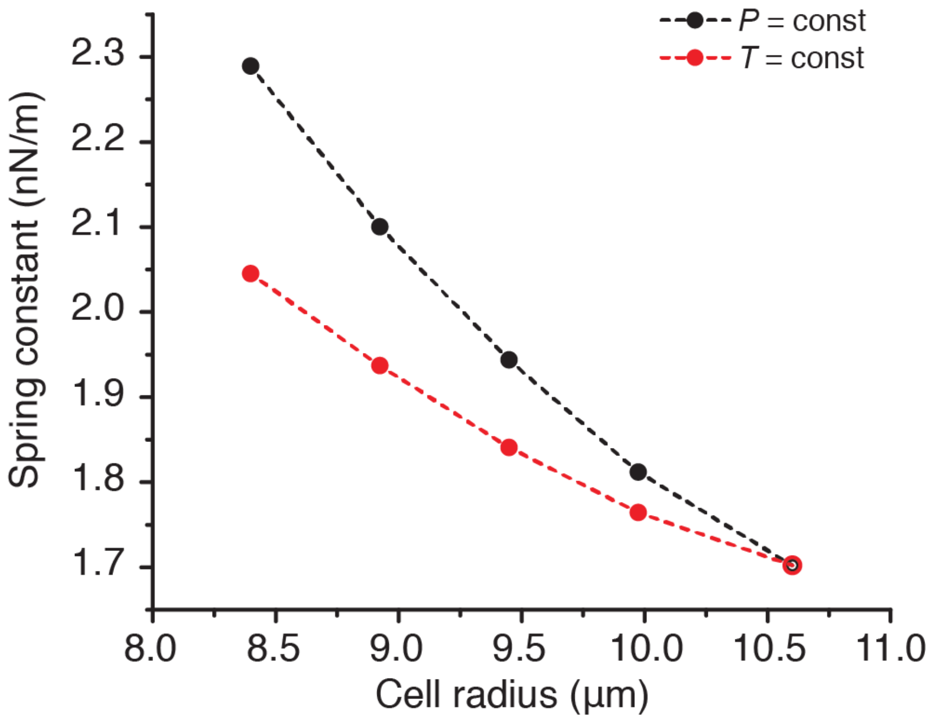

Supplementary Figure 13 | Constant pressure or constant tension show distinguishable cell mechanical properties in dependence of the cell radius. Shown are finite elements simulations of the spring constant of a cell having a total mass of 5 ng, internal pressure (relative to the environment) of $P = 60$ Pa[3] and cortex tension $T = 0.2$ Pa/m[4] (data point with open circle at the right). The filled data points show the spring constant expected for smaller cells having the same tension (T = constant, red) or having the same pressure (P = constant, black).

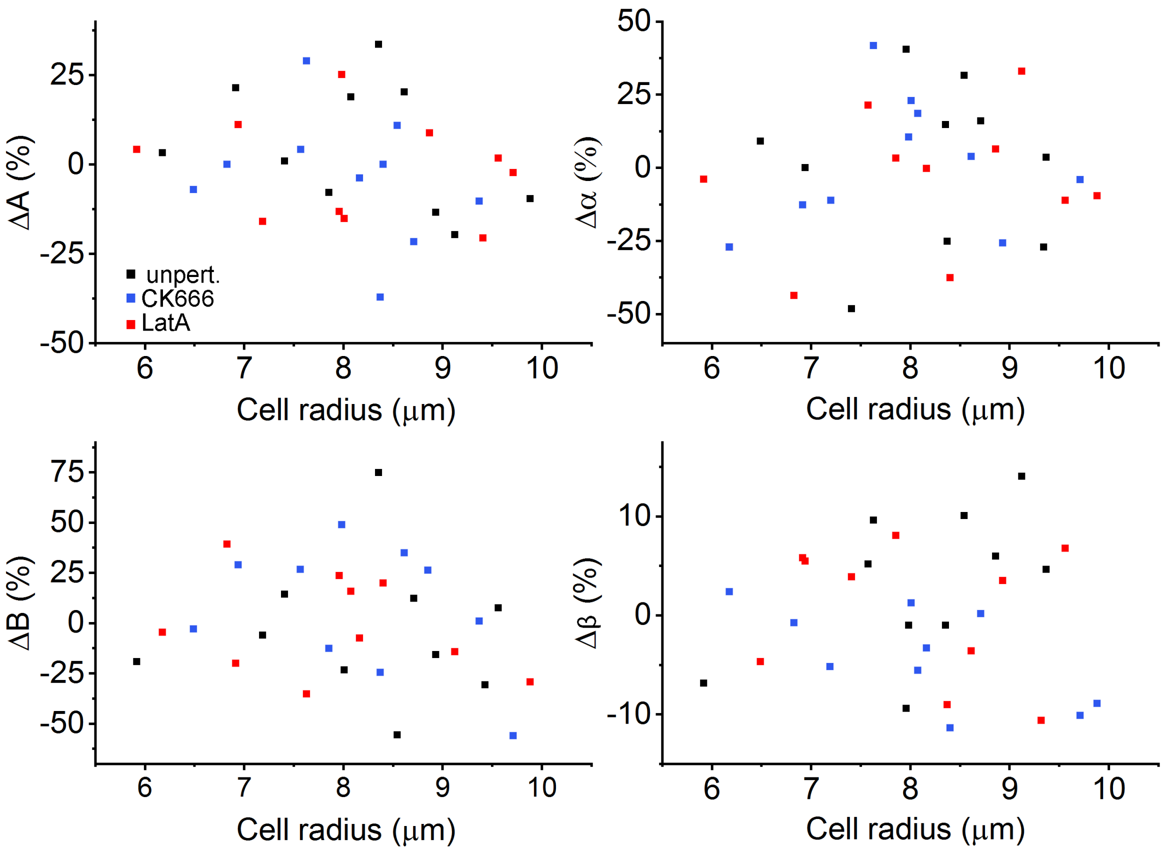

Supplementary Figure 14 | Normalized parameters obtained from fitting the mechanical response of rounded HeLa cells do not correlate with cell radius. The parameters A, B, α, and β used to fit the power-law behavior (Eq. 2) to cell rheology measurements were normalized to the mean and plotted against the cell radius. No significant correlations of cell radii with the individual parameters of the power-law A, B, α, and β were found by applying a Pearson correlation test. Every dot represents one rounded living HeLa cell measured (*n* = 30 biologically different cells). Black dots represent unperturbed HeLa cells, blue dots HeLa cells perturbed with 50 µM CK666, and red dots HeLa cells perturbed with 500 nM LatA.

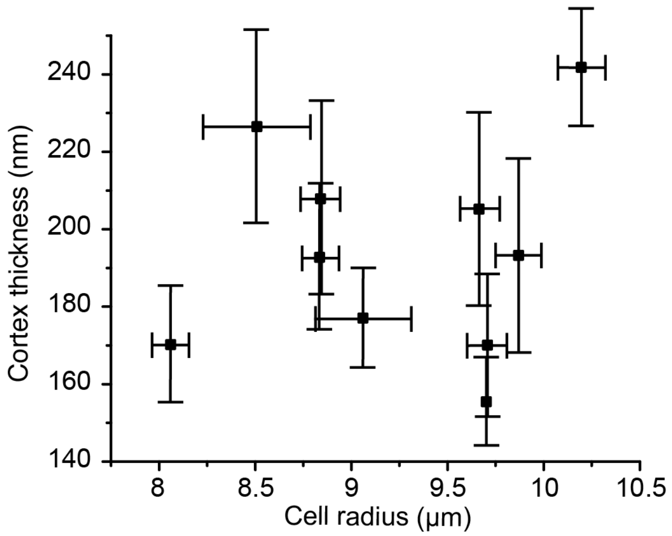

Supplementary Figure 15 | Cortex thickness and diameter of rounded HeLa cells do not correlate. Using super resolution microscopy (STED microscopy), we measured the cortical thickness and diameter of rounded HeLa cells (*n* = 10 biologically different cells). The cortical thickness was determined by averaging the line-width fits of STED images taken from 10 different positions of each cell. The cell diameter represents the average of two orthogonal measurements of the cell diameter. Error bars indicate the standard deviation. A Pearson correlation test showed no significant correlation. Every dot represents one rounded unperturbed HeLa cell measured.

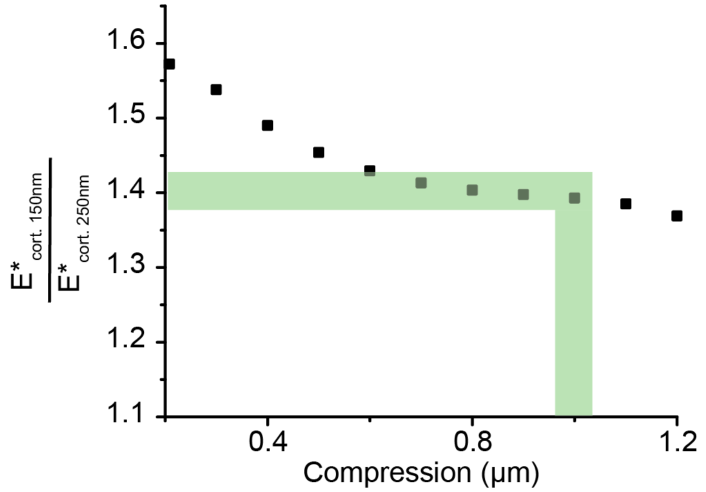

Supplementary Figure 16 | Effect of cellular cortex thickness variation on the complex modulus extraction. The complex modulus $E^*_{\mathrm{cort}} = E'_{\mathrm{cort}} + iE''_{\mathrm{cort}}$ is extracted from $g_c(f)$ under the assumption of a cortex thickness of 200 nm (see **Manuscript** and **Supplementary Note 2)**. However, using STED microscopy on a different setup, we found that the cellular cortex thickness varies between 150 nm and 250 nm (**Fig. 4**) and does not correlate with the cell size (**Supplementary Fig. 15**). To investigate, how this variation affects the extraction of the complex modulus E^*_{cort}, and likewise E'_{cort} and E''_{cort}, we used the solid-shell-liquid-core model (**Supplementary Note 2, Methods**) to simulate a rounded cell with diameter 16 µm, a cortical storage modulus E'_{cort} of 100 kPa, and cortex thicknesses of 250 nm. Under the purposefully wrong assumption of a cortex thickness of 150 nm we then extracted storage moduli. The ratio of the extracted moduli and the real modulus at 150 nm cortex thickness and 100 kPa is shown in the graph against the compression distance Δ. The same dependency holds true for the loss modulus E''_{cort}. As can be seen, assuming a wrong cortex thickness affects the extraction of moduli differently, which depends on Δ. For experimental conditions $\Delta = 1\ \mathrm{\mu m}$, we found a ratio of 1.4, which means that extracting the storage and loss moduli of the cell having a 250 nm thick cortex, but assuming it would be 150 nm thick, leads to overestimate the storage and loss moduli by 40%. In the manuscript, we assume a cortex thickness of 200 nm, which would leads under- and overestimate the cortex moduli by ≈ 20% for cells having a 150 nm and 250 nm thick cortex, respectively. Importantly, the ratio does not change a lot around the experimental conditions used in our work (other than it would change more at smaller compression distances).

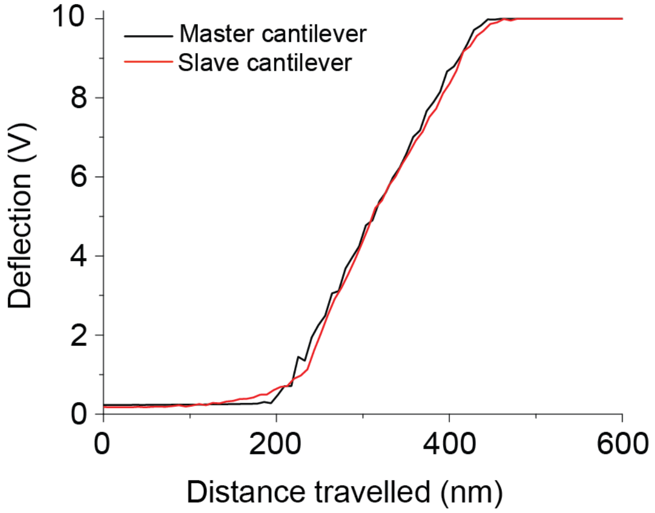

Supplementary Figure 17 | Master and slave microcantilevers show identical deflection sensitivities. The deflection sensitivity of the master microcantilever was determined by touching the Petri dish with the free end and measuring the microcantilever deflection. Thereafter, the laser was shifted to the slave microcantilever. As the slave microcantilever was fixed on the wedge its deflection sensitivity was measured by bringing the support of the master microcantilever into contact with the slave microcantilever. The slave microcantilever shows very similar deflection sensitivity, which is not surprising because both microcantilevers were of the same type and exposed to identical conditions (microcantilever length, width and thickness, laser spot location on the microcantilever, light intensity at the photodiode).

		Unperturbed HeLa cells		CK666 perturbed HeLa cells		LatA perturbed HeLa cells	
Double power-law model	ℓ^{1*}	49125	1.00	44532	1.00	21097	1.00
	ℓ^{2*}	3450	1.00	4203	1.00	1852	1.00
Structural damping model	ℓ^{1*}	50237	1.02	54965	1.23	23297	1.10
	ℓ^{2*}	3478	1.01	4725	1.13	1916	1.04
Single power-law model	ℓ^{1*}	200881	4.09	104306	2.34	71965	3.41
	ℓ^{2*}	9076	2.63	6706	1.59	4093	2.2
Maxwell model	ℓ^{1*}	620292	12.62	411056	9.23	146341	6.94
	ℓ^{2*}	29100	8.43	25533	6.07	8100	4.37

Supplementary Table 1 | The double power-law model describes the experimental data recorded on living HeLa cells best. The single cell storage and loss moduli recorded for unperturbed, CK666 perturbed and LatA perturbed HeLa cells (examples shown in **Fig. 3**) were fitted with four different models: double power-law, structural damping, single power-law, Maxwell (for formulas see caption of **Supplementary Fig. 7** and **Manuscript, Eq. 2**). The fit quality was assessed *via* calculating in two ways how far off the fit is from the experimental data using the distance measures ℓ^{1*} and ℓ^{2*}. The measures differ in how they react to outliers (**Methods**). For each rheological model (row) and experimental condition (column) the two distance measures (top) and the relative distance to the double power-law distance (bottom) are given. The relative distance describes how much better (in this case the relative distance is < 1) or worse (the relative distance is > 1) the respective fit is compared to the double-power law fit. The double power-law fits the experimental data best since the relative distances of all other models are greater than 1. In case of untreated HeLa cells, the structural damping model describes the experimental data almost equally well as the double power-law. In the case of CK666 perturbed HeLa cells, however, the structural damping model describes the experimental data less well. Source data are provided as a Source Data file.

	A [kPa]	α	B [kPa]	β
Unperturbed HeLa cells (*n* = 10)	501 ± 145	0.22 ± 0.04	22 ± 9	0.87 ± 0.05
LatA perturbed HeLa cells (*n* = 10)	113 ± 32	0.21 ± 0.04	20 ± 5	0.86 ± 0.07
Ck666 perturbed HeLa cells (*n* = 10)	278 ± 40	0.14 ± 0.05	90 ± 28	0.62 ± 0.05

Supplementary Table 2 | Parameters of the cell cortex revealed from fitting the power-law behavior of the dynamic modulus to the double power-law. For each HeLa cell rheologically characterized, the function of the frequency dependent, complex modulus $E^*_{\mathrm{cort}} = E'_{\mathrm{cort}} + iE''_{\mathrm{cort}} = A(if/f_0)^{\alpha} + B(if/\,f_0)^{\beta}$ (Eq. 2), was fitted to E'_{cort} and E''_{cort} using a two-dimensional optimization (**Methods**). *n* is the number of biologically different cells. A and B are scaling factors, α and β are low and high frequency exponents, and f_0 is a normalization constant, which has been set to $1\ \mathrm{kHz}$. Values represent the mean and standard deviation. Source data are provided as a Source Data file.

Supplementary Note 1: Derivation of Equation 1

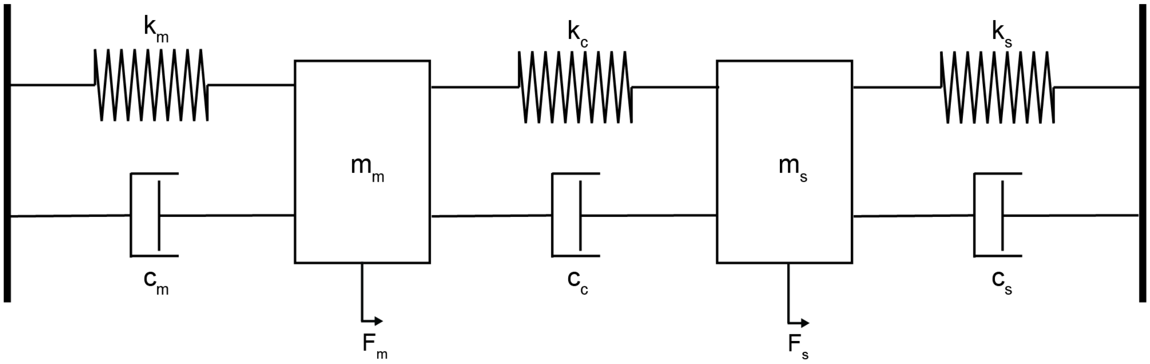

Supplementary Figure 18 | The system of coupled microcantilevers is described as a lumped-mass model. k_m and k_s describe the spring constant and c_m and c_s the damping of the master and slave microcantilevers that are actuated by the photothermal forces F_m and F_s and have the effective masses m_m and m_s, respectively. The coupling of both microcantilevers by the sandwiched cell is modelled with the spring constant k_c and the damping c_c of the cell. The effective mass of each cantilever comprises the mass of the cantilever, the mass of the water dragged by the cantilever, and the mass of the cell adhering to the cantilever.

Derived from the exchange model shown in **Supplementary Fig. 18** We can describe the equation of motions with the matrix notation:

$$M \cdot \begin{pmatrix} \ddot{x}_\mathrm{m} \\ \ddot{x}_\mathrm{s} \end{pmatrix} + C \cdot \begin{pmatrix} \dot{x}_\mathrm{m} \\ \dot{x}_\mathrm{s} \end{pmatrix} + K \cdot \begin{pmatrix} x_\mathrm{m} \\ x_\mathrm{s} \end{pmatrix} = \begin{pmatrix} F_\mathrm{m} \\ F_\mathrm{s} \end{pmatrix} \qquad \text{(Eq. S1)}$$

with

$$M = \begin{bmatrix} m_\mathrm{m} & 0 \\ 0 & m_\mathrm{s} \end{bmatrix},$$

$$C = \begin{bmatrix} c_\mathrm{m} + c_\mathrm{c} & -c_\mathrm{c} \\ -c_\mathrm{c} & c_\mathrm{c} + c_\mathrm{s} \end{bmatrix},$$

$$K = \begin{bmatrix} k_\mathrm{m} + k_\mathrm{c} & -k_\mathrm{c} \\ -k_\mathrm{c} & k_\mathrm{c} + k_\mathrm{s} \end{bmatrix},$$

Hereby, x_m and x_s are the positions of the point masses m_m and m_s, respectively, and the dot notation indicates the total derivatives in time. $k_\mathrm{m}$and k_s describe the spring constant and c_m and c_s the damping of the master and slave microcantilevers that are actuated by the photothermal forces F_m and F_s. Making use of the harmonic drive and the harmonic motion of the microcantilevers we can write Eq. S1 in Fourier space:

$$(\omega^2 M - i\omega C + K) \cdot \boldsymbol{x}(\omega) = \boldsymbol{F}(\omega) \qquad \text{(Eq. S2)}$$

where we define

$$G = (\omega^2 M - i\omega C + K) = \begin{bmatrix} m_\mathrm{m}\omega^2 - i(c_\mathrm{m} + c_\mathrm{c})\omega + k_\mathrm{m} + k_\mathrm{c} & -ic_\mathrm{c} - k_\mathrm{c} \\ -ic_\mathrm{c} - k_\mathrm{c} & m_\mathrm{s}\omega^2 - i(c_\mathrm{c} + c_\mathrm{s})\omega + k_\mathrm{c} + k_\mathrm{s} \end{bmatrix}.$$

We invert and generalize G, writing:

$$\boldsymbol{x}(\omega) = G^{-1}\,\boldsymbol{F}(\omega) = \frac{1}{\det[G]}\begin{bmatrix} g_{22}(\omega) & -g_{12}(\omega) \\ -g_{12}(\omega) & g_{11}(\omega) \end{bmatrix}\begin{pmatrix} F_\mathrm{m} \\ F_\mathrm{s} \end{pmatrix} \qquad \text{(Eq. S3)}$$

where $\left(g_{ij}(\omega)\right) = G(\omega)$, and we made use of the symmetry of the matrix. It is important to note, that the desired coupling properties $(k_\mathrm{c}, c_\mathrm{c})$ are given as the complex spring constant $g_{12}(\omega)$ which can be identified as the cellular transfer function $g_\mathrm{c}(f)$, with $2\pi f = \omega$. If the system can be described as linearly-coupled two masses (terms only linear in x), the off-diagonal elements of G contain only the coupling between m_m and m_s. Therefore, the cellular spring constant and damping, are stored in the diagonal elements of the matrix. This is independent on how the spring constant and damping of the cell are realized, e.g. whether they are described by a power-law, Maxwell or different model. Any complex coupling in the time domain can be expressed by g_{12}. We can think of a complex coupling as a composition of springs and dampers that vary at each frequency. The components of $\boldsymbol{x}(\omega)$ are given by the experiment. Each component's magnitude is given by the measured amplitude and each component's phase is given by the measured phase. To extract g_{12}, we define based on Eq. S3 three different functions: $\chi_\mathrm{MM}(\omega) = F_\mathrm{m} g_{22}/\det[G]$, which is given by the position the mass m_m when $F_\mathrm{s} = 0$, $\chi_\mathrm{MS}(\omega) = -F_\mathrm{m} g_{12}(\omega)/\det[G]$, which is given by the position mass m_s when $F_\mathrm{s} = 0$, and $\chi_\mathrm{SS}(f) = F_\mathrm{s} g_{11}(\omega)/\det[G]$, which is given by the position mass m_s when $F_\mathrm{m} = 0$. Combining the three functions, we write:

$$\chi_\mathrm{MM}(\omega) \cdot \chi_\mathrm{SS}(\omega) - n \cdot \chi_\mathrm{MS}(\omega) = \frac{F_\mathrm{s}^2 \cdot n}{\det[G]} \qquad \text{(Eq. S4)}$$

where $n = F_\mathrm{s}/F_\mathrm{m}$. By solving this expression for $\det[G]$, we extract g_{12} from $\chi_{MS}(\omega)$ as:

$$g_{12}(\omega) = \frac{\chi_\mathrm{MS}(\omega)}{\chi_\mathrm{MM}(\omega) \cdot \chi_\mathrm{SS}(\omega) - F_\mathrm{m}/F_\mathrm{s}\, \chi_\mathrm{MS}^2(\omega)} F_\mathrm{m} \qquad \text{(Eq. S5)}$$

With $\omega = 2\pi f$, we can replace $g_{12}(\omega)$ with $g_c(f)$ and obtain Eq. 1 of the manuscript.
For the application of Eq. S5 the actual values for m_m and m_s are not of importance as they are contained in the three measured functions $\chi_\mathrm{MM}, \chi_\mathrm{MS}, \chi_\mathrm{SS}$. Therefore, also the distribution of the cell mass across the cantilevers does not need to be known.

Supplementary Note 2: Extraction of E'_{cort} and E''_{cort} from Eq. 1

Eq. 1 yields the frequency dependent, complex cellular transfer function $g_c(f)$, which is a complex spring constant stemming from the spatially averaged mechanical properties of the entire, single cell. In order to convert the geometry dependent $g_c(f)$ into a geometry independent, dynamic modulus $E^*_{\mathrm{cort}}(f) = E'_{\mathrm{cort}}(f) + iE''_{\mathrm{cort}}(f)$, we introduce a shape factor *S*: $g_c(f) = S \cdot E^*_{\mathrm{cort}}(f)$. S captures all geometry dependencies of $g_c(f)$ whereas $E^*_{\mathrm{cort}}(f)$ captures all geometry-independent, cortex-material dependencies. To find S we employed finite-elements modelling. Using a solid shell liquid core model (**Methods, Supplementary Fig. 19a**), we found the relationship between the compression distance Δ and real part of the cellular transfer function g'_c for a fixed frequency, i.e. $\mathrm{Re}(E^*_{\mathrm{cort}}(f)) = E'_{\mathrm{cort}}(f) = E'_{\mathrm{cort}}$. Hereby, g_c' is in the units of a spring constant. Several of these spring constant *versus* compression distance curves are shown in **Supplementary Fig. 19b**. We then made a third order polynomial ansatz for *S*:

$$g'_c = E'_{\mathrm{cort}} \cdot S = E'_{\mathrm{cort}} \cdot h_{\mathrm{cort}}(a_1\Delta/R + (a_2\Delta/R)^2 + (a_3\Delta/R)^3) \qquad \text{(Eq. S6)}$$

where h_{cort} is the thickness of the cell cortex and *R* the radius of the uncompressed cell. To find a_1, a_2, a_3, we fitted Eq. S6 to a single spring constant *versus* compression distance curve that was generated with $E'_{\mathrm{cort}} = 125$ kPa and $R = 8.4$ µm. This radius corresponds to a cell with mass of 2.5 ng. We found $a_1 = 15 \cdot 10^{-12}, a_2 = 9.7 \cdot 10^{-12}, a_3 = 5 \cdot 10^{-12}$. The three parameters were then used to extract E'_{cort}. from the spring constant versus compression distance curves (**Supplementary Fig. 19b,c**) according to $E'_{\mathrm{cort}} = g'_c/(h_{\mathrm{cort}}(a_1\Delta/R + (a_2\Delta/R)^2 + (a_3\Delta/R)^3)$. We successfully extracted different E'_{cort} for a fixed R, (**Supplementary Fig. 2b**). This means that that the parameters a_1, a_2, a_3 found for $E'_{\mathrm{cort}} = 125$ kPa are valid for a wide range of E'_{cort}, as encountered in the experiment. Also, we successfully extracted E'_{cort} for varying R and a fixed E'_{cort} (**Supplementary Fig. 19c**). This means that that the parameters a_1, a_2, a_3 found for $R = 8.4$ µm are valid for a wide range of R, as encountered in the experiment. It is important to note that the geometry of the cell, and hence the shape factor *S* of the cell, is determined by the initial cell radius and the height of the compressed cell. The cell radius R is extracted *via* the cell mass measurement, whereas Δ can be accurately chosen *via* the microcantilever distance during the experiment. The contact area of the cell and a sandwiching cantilever (**Supplementary Fig. 19a**), is not necessary to be measured as it results from compressing a spherical cell of radius R by the distance Δ and is therefore already captured in the model. The same S is used in Eq. S6 to extract E''_{cort} according to $E^*_{\mathrm{cort}}(f) \cdot S = (E'_{\mathrm{cort}} + iE''_{\mathrm{cort}}) \cdot S = g_c(f)$.

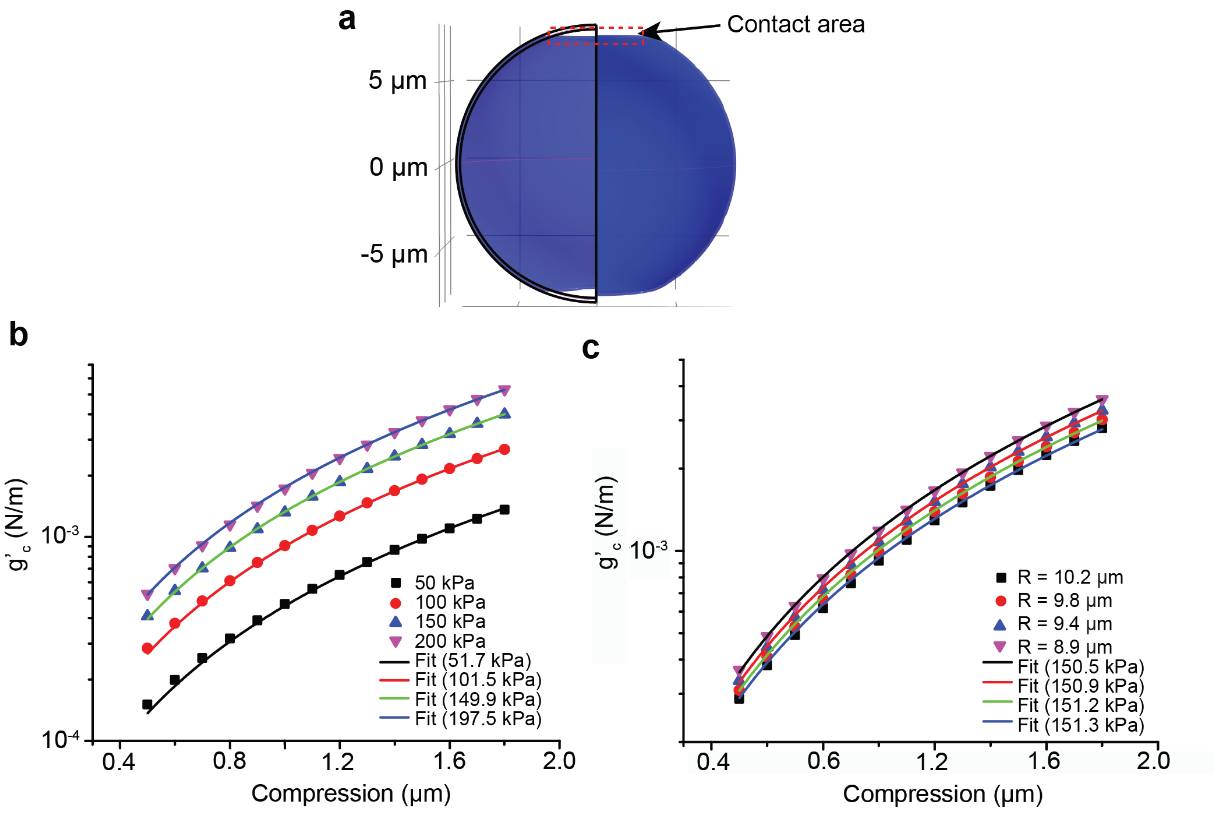

Supplementary Figure 19 | Extracting E'_{cort} from the real part cellular transfer function $\mathrm{Re}(\boldsymbol{g_c}) = \boldsymbol{g'_c}$. **a**, To extract the storage modulus from g'_c, we simulated conditions in which a solid-shell-liquid-core cell with a cortical thickness of h_{cort} =200 nm (**Fig. 4f**), varying size and cortical storage modulus is compressed by the distance Δ. **b,** g'_c simulated for a rounded cell having E'_{cort}a cell radius of $R = 8.4$ μm, and having storage moduli E'_{cort} of 50, 200, 150 or 200 kPa. **c,** g'_c calculated for a rounded cell having a cortical storage modulus of 150 kPa and having radii *R* of 8.9, 9.4, 9.8 and 10.2 μm corresponding to cell masses of 3, 3.5, 4 and 4.5 ng, respectively.

Supplementary Note 3: Tension dependency of storage modulus E'_{cort}

To consider the dependency of the dynamic modulus $E^*_{\mathrm{cort}} = E'_{\mathrm{cort}} + iE''_{\mathrm{cort}}$ with respect to cell cortex tension T and pressure P , it is illustrative to consider that the storage modulus E'_{cort} is the ratio of the energy stored U per cell cortex volume V_{cort}, $E'_{\mathrm{cort}} = U/V_{\mathrm{cort}}$. Upon compressing the rounded cell by the distance Δ, each point of the cell cortex displaces by $\delta(\Delta)$ and we can write: $U = T\int(\frac{1}{R}d\delta/d\Theta)^2 dA_c$ where Θ is the meridian angle and R the cell radius (**Supplementary Fig. 20**). With $d\delta/d\Theta \approx 2\Delta/\pi$ we can approximate the integral to be $U \approx \left(\frac{8}{\pi}\right)\Delta^2 T$. Thus, the energy stored in the cell cortex is proportional to the cortex tension T and quadratic to the compression distance Δ. The volume of the cell cortex having a thickness h_{cort} corresponds to $V_{\mathrm{cort}} = 4\pi R^2 h_{\mathrm{cort}}$. Thus, the storage modulus is proportional to T/R^2 or if expressed in terms of pressure using the Laplace law $E'_{\mathrm{cort}} \approx P/R$.

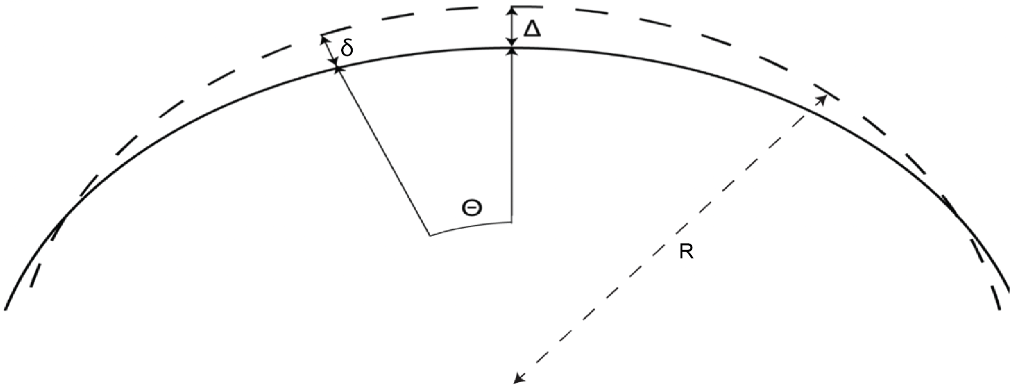

Supplementary Figure 20 | Geometry of a cell compressed by a small distance. Upon compressing a rounded cell of radius R (dashed line) by the distance Δ, the cell deforms (continuous line). δ is the radial displacement of every point of the cortex at the meridian angle Θ. By analogy of a string under tension, we can calculate the energy U needed to deform the cell.

SUPPLEMENTARY INFORMATION REFERENCES

1 Ramos, D., Tamayo, J., Mertens, J. & Calleja, M. Photothermal excitation of microcantilevers in liquids. *J. Appl. Phys.* **99**, (2006).

2 Rigato, A., Miyagi, A., Scheuring, S. & Rico, F. High-frequency microrheology reveals cytoskeleton dynamics in living cells. *Nat. Phys.* **13**, 771-775, (2017).

3 Fischer-Friedrich, E., Hyman, A. A., Julicher, F., Muller, D. J. & Helenius, J. Quantification of surface tension and internal pressure generated by single mitotic cells. *Sci. Rep.* **4**, (2014).

4 Cartagena-Rivera, A. X., Logue, J. S., Waterman, C. M. & Chadwick, R. S. Actomyosin Cortical Mechanical Properties in Nonadherent Cells Determined by Atomic Force Microscopy. *Biophys. J.* **110**, 2528-2539, (2016).
